# Investigation on the Preparation of Rice Straw-Derived Cellulose Acetate and Its Spinnability for Electrospinning

**DOI:** 10.3390/polym13203463

**Published:** 2021-10-09

**Authors:** Juntao Yan, Jinhong Liu, Ya Sun, Guangsen Song, Deng Ding, Guozhi Fan, Bo Chai, Chunlei Wang, Linbing Sun

**Affiliations:** 1 College of Chemistry and Environmental Engineering, Wuhan Polytechnic University, Wuhan 430023, China; 12112@whpu.edu.cn (J.L.); sunya230@whpu.edu.cn (Y.S.); 12662@whpu.edu.cn (G.S.); dingdeng@whu.edu.cn (D.D.); fgzcch@whpu.edu.cn (G.F.); chaibo@whpu.edu.cn (B.C.); 2 College of Chemical Engineering, Nanjing Tech University, Nanjing 211816, China; lbsun@njtech.edu.cn

**Keywords:** rice straw-derived cellulose, cellulose acetate, electrospinning, spinnability

## Abstract

Rice straw-derived cellulose (RSC) with purity of 92 wt.% was successfully extracted from rice straw by a novel and facile strategy, which integrated the C_2_H_5_OH/H_2_O autocatalytic process, dilute alkali treatment and H_2_O_2_ bleaching process. Influencing factors of the cellulose extraction were systematically examined, such as ethanol concentration, alkali concentration, H_2_O_2_ bleaching process and so on; the optimal extraction conditions of cellulose was determined. A series of rice straw-derived cellulose acetate (RSCA) with different degree of substitution (DS) were prepared by the acetylation reaction; the effects of Ac_2_O/cellulose ratio, reaction temperature and reaction time on the acetylation reaction were investigated. Results of FTIR and XRD analysis demonstrated that highly purified RSC and RSCA were prepared comparing with the commercial cellulose and cellulose acetate. Solubility analysis of RSCA with different DS indicated as-prepared RSCA with DS of 2.82 possessed the best solubleness, which was suitable for electrospinning. Moreover, the flexible RSCA fibrous membrane was easily fabricated by a facile electrospinning method. Our proposed method provided a strategy for realizing the high-value utilization of waste rice straw resource, as prepared RSC and RSCA can be used as chemical raw material, and electrospun RSCA fibrous membrane has various applications in medical materials, food packaging, water purification and so on.

## 1. Introduction

Crop straw is rich in resources and extensive in use, and annual output is about 900 million tons in China. Therefore, it is advantageous to utilize waste crop straw to alleviate the shortage of forest and fossil resources [1], but if not effectively used, it will become a huge source of pollution, bringing about environmental pollution [2]. Rice straw is an important by-product of crops; with the enhancement of people’s awareness of using crop waste resources [3,4,5], more and more researchers are paying attention to the comprehensive utilization of rice straw resources. Methods of utilization are mainly focused on energy supply by incineration [6], landfill compost [7], livestock feed [8], etc. Comprehensive utilization of rice straw is beneficial for alleviating the energy storage problem, stabilizing agricultural ecological balance and solving the pollution problem [9]. As it is known that rice straw is mainly composed of cellulose (40–50%), hemicellulose (20–30%) and lignin (10–18%) [10], cellulose is trapped in a hemicellulose-lignin matrix, which makes its separation extremely difficult [11,12]. Moreover, a complex hydrogen bond network exists among the cellulose molecules; thereby, cellulose is insoluble in common solvents. At present, inorganic acid-base cooking [13] and organic solvent extraction are the major methods for extracting cellulose from crop straw. For example, Guo et al. put forward a method using a thermal acetic acid/sodium acetate hydrolysis process to separate cellulose pulp by regulating the molar ratio of acetic acid to sodium acetate; the separated cellulose pulp has high intrinsic viscosity and high glucan content. However, some hemicelluloses and lignin remain in the cellulose pulps. The residual xylan, mannan and lignin in the cellulose pulp were 17.19%, 1.24% and 8.91%, respectively [11]. Wang et al. extracted cellulose from fruit pomace by heating it in a HCl solution and NaOH solution successively and, then, bleached it with sodium hypochlorite [14]. Glinska et al. extracted the cellulose from corn stover using designed ionic liquids with improved reusing capabilities; the synthesized ionic liquids turned out to dissolve the greatest amount of cellulose (84%) [15].

In addition to the above methods, there is also microwave-assisted extraction [16] and steam explosion pretreatment methods, but most of the methods utilize a large number of acid and alkali, which results in serious damage to the environment or has the disadvantage of complex experimental procedures and high cost; moreover, the above process of extraction will break the structure of hemicellulose and lignin. Nevertheless, the solvent of ethanol can effectively dissolve lignin and separate cellulose and hemicellulose [16]. For example, Rehman et al. extracted lignocellulose from crude corn straw by employing the mixed solvent of n-hexane, ethanol and deionized water, followed by a multi-step process of weak alkali treatment and complete chlorine-free bleaching. Rosa et al. successfully separated cellulose from rice husk by means of a chlorine-free procedure, on the basis of the whole cellulose content expected for rice husk; this method resulted in a yield around 74%, in which the effect of ethanol was also examined [17].

Cellulose, the most common natural polymer on earth, is renewable, inexpensive and widely available, attracting wide interest in the biomedical, food packaging and pharmaceutical industries [18,19,20]. Cellulose is not only used to produce nano cellulosic materials for biomedical, energy and electronics applications [21], but can also be converted into the biofuel of cellulosic ethanol, which becomes an important alternative fuel source [22]. In addition, cellulose can be converted into nutrients such as glucose for human use [23]. Cellulose acetate that is synthesized via the acetylation reaction of cellulose is an important cellulose derivative, which possesses the advantages of good biocompatibility and environmental friendliness. Cellulose acetate has great potential for electrospinning; it can be easily prepared into fibers or thin film materials, which can be applied in the field of water pollution control and purification [24].

Generally speaking, cellulose is difficult to spin by electrospinning [25]; however, cellulose acetate possesses good spinnability for electrospinning, which depends on the following two aspects. On the one hand, the premise is that its precursor of cellulose has high purity with very little insoluble matter. On the other hand, the cellulose acetate possesses a suitable degree of substitution.

In this paper, we develop a novel and facile strategy for realizing the high-value utilization of waste rice straw resource, which integrates the C_2_H_5_OH/H_2_O autocatalytic process, dilute alkali treatment and H_2_O_2_ bleaching process. Rice straw-derived cellulose (RSC) with a purity of 92 wt.% and a series of rice straw-derived cellulose acetate (RSCA) with different degree of substitution (DS) are achieved, and the optimal process conditions are determined. Moreover, the RSCA suitable for electrospinning with a DS value of 2.82 is screened out, and the electrospun RSCA fibrous membrane is successfully fabricated, which has various applications in medical materials, food packaging, water purification and so on.

## 2. Materials and Methods

### 2.1. Materials

Rice straw was obtained from Hubei province, China. Absolute C_2_H_5_OH, CH_2_Cl_2_, CH_3_OH, hydrogen peroxide (H_2_O_2_), acetic anhydride (Ac_2_O), acetic acid (HAc, 99.5%), concentrated sulfuric acid (H_2_SO_4_) and potassium hydroxide (KOH) were purchased from Sinopharm Group Chemical Reagent Co. LTD. (Shanghai, China) Poly(vinyl pyrrolidone) (PVP, Mw∼1,300,000), commercial cellulose and commercial cellulose acetate were purchased from Aladdin Regent Company. (Shanghai, China).

### 2.2. Rice Straw-Derived Cellulose Extraction

**Figure 1:**
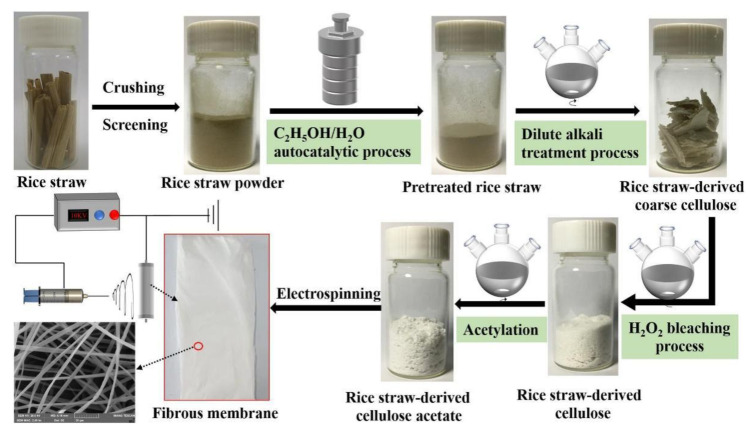
Schematic illustration of the preparation and electrospinning of rice straw-derived cellulose acetate.

Cellulose was extracted from rice straw by a facile and novel method, which integrated the C_2_H_5_OH/H_2_O autocatalytic process, dilute alkali treatment process and H_2_O_2_ bleaching process; the schematic illustration of the extraction of cellulose from rice straw is proposed in Figure 1. Firstly, the rice straw was washed, dried and pulverized by a pulverizer (BJ-800A, Baijie Crusher Machinery Co., Zhejiang, China), then the 80–100 mesh rice straw powder was sieved for use. Rice straw powder was pretreated as follows: 5 g rice straw powder and a certain amount of C_2_H_5_OH/H_2_O with different concentration were charged into a 100 mL Teflon-lined stainless steel autoclave at 200 °C for 2.5 h and, then, cooled to the temperature. The pretreated rice straw powder and black liquor were obtained by the suction filtration, respectively. Then, the pretreated rice straw powder was washed by the same concentration of C_2_H_5_OH/H_2_O until the filtrate was clarified, and the wash filtrate was collected together. The dissolved lignin and adsorbed lignin were achieved by adding triploid volume H_2_O into the black liquor and wash filtrate, respectively. The pH value of black liquor was measured. The quality of dissolved lignin and adsorbed lignin were weighted after the drying, and the lignin was furtherly used in other studies. The mass of pretreated rice straw powder was weighted after the drying. Secondly, the above pretreated rice straw powder was confronted with dilute alkali treatment. Specifically, 2 g pretreated rice straw and 40 mL KOH aqueous solution with a different concentration were added to the round bottom flask and stirred at 90 °C for 2 h; subsequently, the precipitate of coarse cellulose was filtered out, and the yield of coarse cellulose was calculated on the basis of 2 g pretreated rice straw. Finally, the achieved coarse cellulose was confronted with the H_2_O_2_ bleaching processes, and coarse cellulose and 200 mL 2 wt.% H_2_O_2_ solution were heated at 70 °C for 2 h to obtain the rice straw-derived cellulose (RSC).

### 2.3. Preparation of Rice Straw-Derived Cellulose Acetate

Rice straw-derived cellulose acetate (RSCA) was prepared by the acetylation on the basis of RSC. To be specific, 0.5 g rice straw-derived cellulose, 8 mL HAc, a certain amount of concentrated H_2_SO_4_ and Ac_2_O a were added to three-necked, round-bottomed flask for acetylation at a certain temperature. After the reaction time finished, distilled water was added to the acetylated solution for precipitation, and then, vacuum filtration was carried out. Deionized water was used to wash the above-mentioned precipitation until the filtrate was neutral. Finally, the samples were freeze-dried to obtain the RSCA.

### 2.4. Fabrication of the RSCA Fibrous Membrane

A certain volume of dichloromethane (CH_2_Cl_2_) and glacial acetic acid (HAc) (*v*/*v* = 2/1) were stirred and mixed to form homogeneous solution; then, a certain quality of CA and PVP was charged into the above solution, followed by magnetic stirring for 6 h to obtain the spinning solution of CA/PVP (*w*:*w* = 8:2). The content of CA/PVP was fixed at 12 wt.%. Herein, PVP was employed as the spinning additive, which could facilitate the electrospinning. Finally, the spinning solution was loaded into a syringe equipped steel needle, the collector was placed 15 cm away from the tip of steel needle, and the temperature and humidity of spinning were set at 25–27 °C and about 40%, respectively. A negative voltage of −5 kV and positive voltage of 10 kV were supplied by a commercial electrospinning equipment (ET-3556H, Ucalery. Co. Ltd., Beijing, China); the RSCA fibrous membrane was obtained by electrospinning.

### 2.5. Determination of the Cellulose Content and DS Value of RSCA

The cellulose content in the sample and DS value of cellulose acetate were determined according to the method reported in the references [26,27].

### 2.6. Determination the Insoluble Matter of RSCA

An amount of 0.50 g RSCA powder and 20 mL CH_2_Cl_2_/CH_3_OH (*v*/*v* = 9:1) were added to the conical flask and stirred for 100 min on a magnetic stirrer (HWJB-2100A, Zhengzhou Carbon Bang Instrument Co. Ltd., Zhengzhou, China), which was centrifuged for 30 min at a speed of 4500 r/min to separate the supernatant. N-hexane was added to the supernatant liquid and the lower solid layer, respectively. The precipitation was washed with C_2_H_5_OH and, then, freeze-dried. The quality of insoluble matter of RSCA was obtained. The formula in reference [28] could be used to determine the insoluble matter content in cellulose acetate.

### 2.7. Characterizations

The FTIR spectrum was measured by NEXUS 670 Fourier Transform Infrared Spectroscopy from Thermo Nicolet (Shanghai, China) in the range of 4000–500 cm^−1^, and KBr was compressed into a pellet. The crystal phases of samples were measured by powder X-ray diffraction (XRD, Shimadzu XRD-7000 diffractometer, Shanghai, China) using Cu Ka irradiation at 40 kV and 30 mA. The scanning range was 2θ = 10~60°. The surface morphologies of samples were examined using scanning electron microscopy (SEM, TESCANMAIA 3 LMH, TESCAN Company, Czech Republic).

## 3. Results

### 3.1. Optimization of the Extraction Conditions of RSC

**Figure 2:**
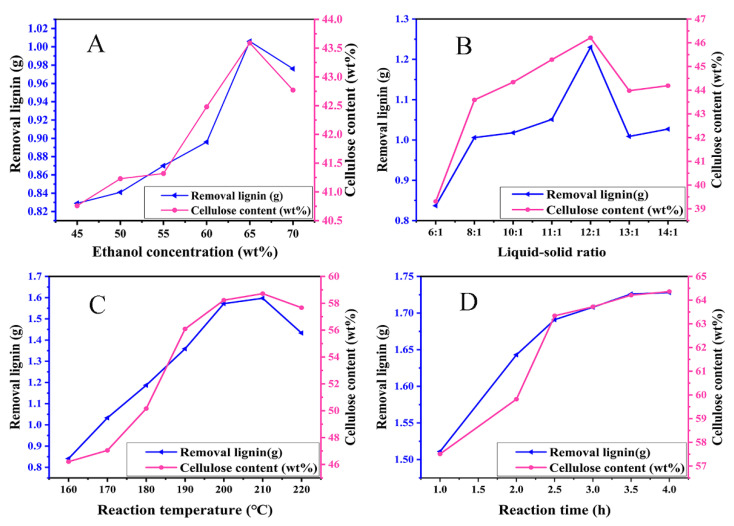
Optimization of the extraction conditions of RSC: (**A**) ethanol concentration, (**B**) liquid–solid ratio, (**C**) reaction temperature, (**D**) reaction time.

The extraction of cellulose from rice straw was dependent on the C_2_H_5_OH/H_2_O autocatalytic process, dilute alkali treatment process and H_2_O_2_ bleaching process, which integrated the facile method for the cellulose extraction. For the C_2_H_5_OH/H_2_O autocatalytic process of rice straw, the effects of ethanol concentration, liquid–solid ratio, reaction temperature and reaction time on the cellulose extraction were systematically examined. As can be seen from Figure 2A, when the C_2_H_5_OH concentration increased from 45 to 70 wt.%, the amount of removal lignin first increased and, then, decreased; meanwhile, the variation of cellulose content in the pretreatment rice straw showed a similar trend, and the pH of the reaction system was in the range of 5.36–5.59, which was ascribed to the production CH_3_COOH during the C_2_H_5_OH/H_2_O autocatalytic process. For 5 g rice straw powder, when the C_2_H_5_OH concentration was 65 wt.%, the amount of removal lignin was a maximum of 1.006 g; simultaneously, the cellulose content in the pretreatment rice straw also reached the maximum of 43.59 wt.%. The reasons are attributed to the following two aspects: On the one hand, the content of H_2_O in the solvent system decreased with the increasing C_2_H_5_OH concentration, which resulted in the decrease in thermal capacity and energy absorbed of the solvent system; thus, it is not conductive to the dissolution of lignin from the rice straw [29]. On the other hand, when the C_2_H_5_OH concentration exceeded 65 wt.%, more CH_3_COOH was produced, and the esterification reaction between C_2_H_5_OH and CH_3_COOH was easily reacted, which was not helpful for the catalytic cracking of ether bonds of lignin; therefore, the dissolution of lignin from rice straw was reduced [30].

The number of active groups and the concentration of reactants in the reaction system were affected by the liquid–solid ratio of C_2_H_5_OH/H_2_O to rice straw powder. Therefore, it is necessary to optimize the liquid–solid ratio. As presented in Figure 2B, the amount of removal lignin increased firstly and, then, decreased, and the content of cellulose also increased firstly and, then, decreased as the liquid–solid ratio increased; moreover, the pH of the reaction system was in the range of 5.32–5.45. When the liquid–solid ratio was 12:1, the removal amount of lignin was the largest, and the cellulose content of pretreated rice straw also reached the maximum of 46.21%. After continuing to increase the liquid–solid ratio, both the removal amount of the lignin and the cellulose content were decreased. When the liquid–solid ratio was low, the number of active groups in the solvent was few, and the contact between the solvent and rice straw powder was not sufficient; moreover, the thermal capacity of the solvent system was so low that the pyrolysis and dissolution of lignin was weakened [31]. When the liquid–solid ratio was too large, the excessive C_2_H_5_OH was prone to esterify with CH_3_COOH produced by the C_2_H_5_OH/H_2_O autocatalytic process, which was not conducive to removing lignin from rice straw.

As seen from Figure 2C, it is observed that the reaction temperature of the C_2_H_5_OH/H_2_O autocatalytic process had an important influence on the amount of removal lignin. With the increase in reaction temperature from 160 to 220 °C, the removal amount of lignin increased firstly and, then, decreased, and the cellulose content of pretreated rice straw had a similar trend. When the reaction temperature was 210 °C, both the removal amount of lignin and the cellulose content of pretreated rice straw reached the maximum of 1.597 g and 58.72%, respectively. Especially, when the reaction temperature increased from 200 to 210 °C, both the removal amount of lignin and the cellulose content of pretreated rice straw had a slight change; this was because lignin and cellulose began to degrade when the temperature exceeded the 200 °C [32]. As a result, the reaction temperature for the C_2_H_5_OH/H_2_O autocatalytic process was determined to be 200 °C.

Other experimental conditions were kept constant, and the reaction time was optimized. As seen from Figure 2D, when the reaction time was prolonged, the removal amount of lignin and the cellulose content of pretreated rice straw first increased rapidly and, then, increased slightly, especially when the reaction time exceeded 2.5 h. Furthermore, the pH of the reaction system varied in the range of 4.58–4.80. Therefore, the reaction time for the C_2_H_5_OH/H_2_O autocatalytic process was determined to 2.5 h.

**Figure 3:**
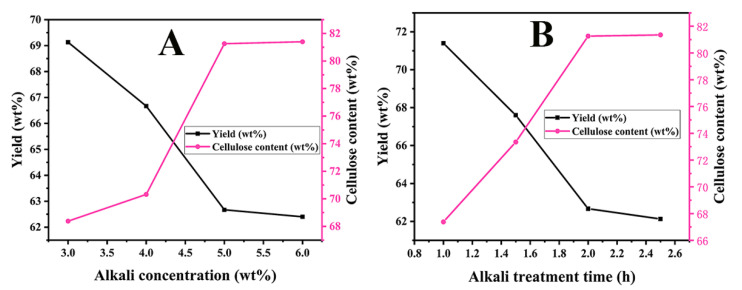
Optimization of the dilute alkali treatment process: (**A**) alkali concentration, (**B**) alkali treatment time.

For the dilute alkali treatment process, the influences of alkali concentration and treatment time on the coarse cellulose extraction were investigated. During the dilute alkali treatment process for the pretreated rice straw, different KOH concentrations were examined. As seen from Figure 3A, the yield of as-prepared coarse cellulose decreased and the cellulose content increased with the increasing concentration of KOH, which indicated that the high KOH concentration could furtherly remove lignin from the coarse cellulose; herein, the lower the yield, the more lignin was removed and the higher the cellulose content. When KOH concentration was 5 wt.%, the product yield and cellulose content were 62.67 wt.% and 81.26 wt.%, respectively. However, the variation of product yield and cellulose content were negligible when the KOH concentration exceeded 5 wt.%. It is suggested that 5 wt.% KOH aqueous solution was sufficient for removing most lignin from the pretreated rice straw. Moreover, the alkali treatment time was also investigated; as depicted in Figure 3B, the product yield decreased and the cellulose content increased when the alkali treatment time was prolonged. However, the changes were tiny when the alkali treatment time exceeded 2 h. Finally, the as-prepared coarse cellulose was confronted with the H_2_O_2_ bleaching process to enhance the cellulose content, which was improved from the 81.26 to 92 wt.%, and the refined cellulose was achieved after the H_2_O_2_ bleaching process. Among references, only references [21] and [33] reported the content of cellulose, which was 85.31 ± 0.91% and 71%, respectively.

### 3.2. Optimization of the Preparation Conditions of RSCA

**Figure 4:**
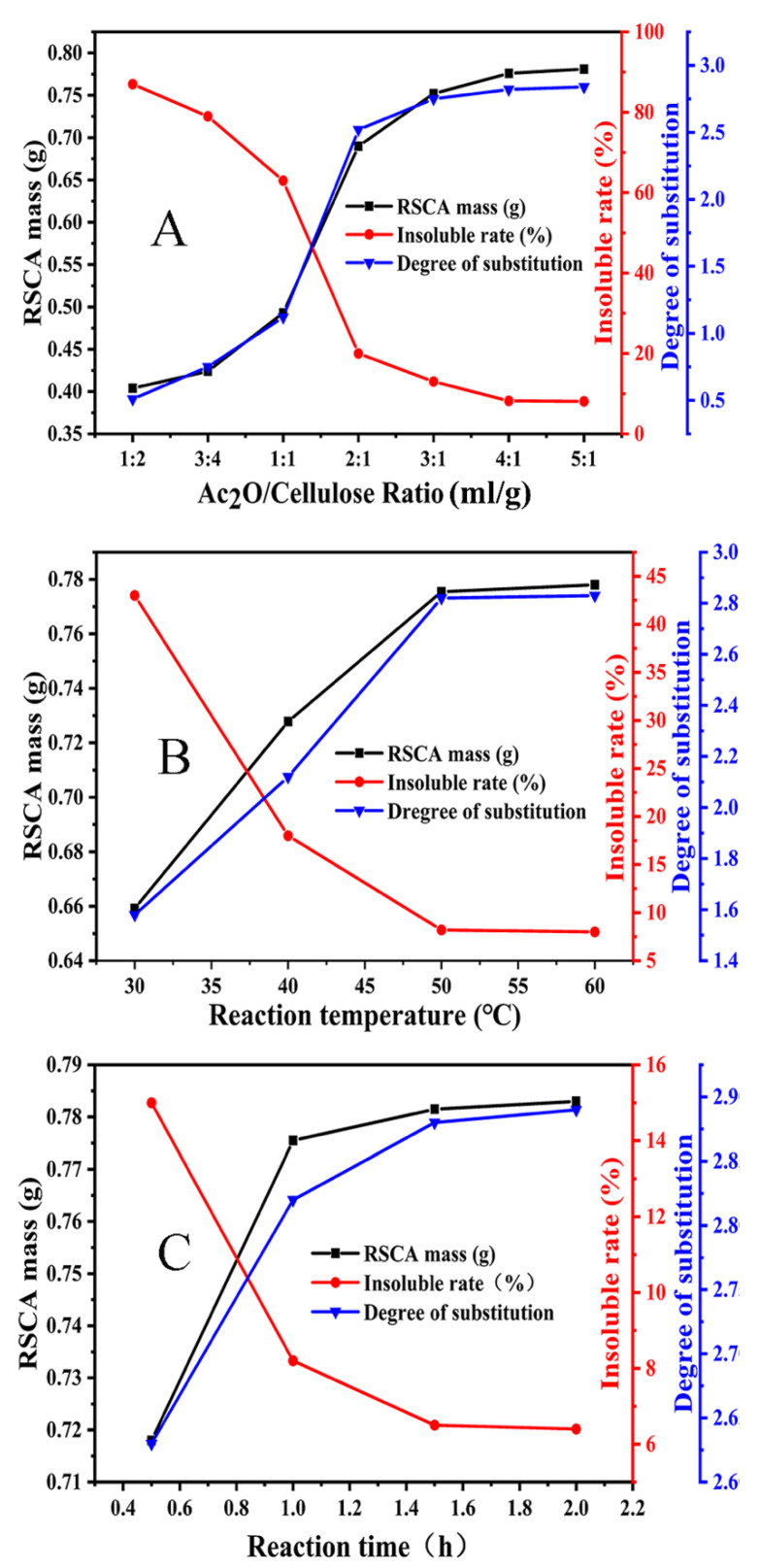
Optimization of the preparation conditions of RSCA: (**A**) Ac_2_O/cellulose ratio, (**B**) reaction temperature, (**C**) reaction time.

The effects of the Ac_2_O/cellulose ratio, reaction temperature and reaction time on the acetylation reaction were examined in detail. As seen from Figure 4A, when the Ac_2_O/cellulose ratio increased, in other words, the Ac_2_O dosage increased; as a result, both the mass and DS of RSCA gradually increased. However, the insoluble rate of RSCA markedly decreased, and the change in insoluble rate was tiny when the Ac_2_O/cellulose ratio exceeded 4:1; thus, the Ac_2_O/cellulose ratio of 4:1 was determined. The acetylation reaction was a reversible reaction, and the increment of Ac_2_O dosage was conductive to promoting the reaction equilibrium towards the esterification; therefore, both the mass and degree of substitution of RSCA gradually increased [34].

In Figure 4A, seven RSCA samples with different DS were prepared when the Ac_2_O/cellulose ratio were changed. When the Ac_2_O/cellulose ratio was 1:2, 3:4, 1:1, 2:1, 3:1, 4:1 and 5:1, RSCA samples with DS of 0.51, 0.75, 1.12, 2.52, 2.75, 2.82 and 2.84 were achieved, respectively. Five RSCA samples with DS of 0.51, 0.75, 1.12, 2.75 and 2.82 were selected to examine the solubility in methanol/dichloromethane solution, the insoluble mass was separated and weighted, and the insoluble rate was calculated to be 87%, 79%, 63%, 13% and 8.2%, correspondingly, which decreased with the increasing DS. The results of insoluble rate were consistent with the results shown in Figure 8B; specifically, the observed precipitation at the bottom gradually diminished.

As seen from Figure 4B, the mass and DS of RSCA gradually increased with the increasing reaction temperature; on the contrary, the insoluble rate of RSCA obviously decreased. However, the changes were negligible when the reaction temperature exceeded 50 °C; thus, 50 °C was chosen as the optimal temperature of acetylation reaction. The increasing temperature facilitated the acetylation reaction between the (Ac)_2_O and hydroxyl groups of cellulose [35]. Therefore, the mass and substitution degree of RSCA increased with the increasing reaction temperature.

It is shown in Figure 4C that the mass and DS of RSCA raised with the extension of reaction time, but the insoluble rate of RSCA dramatically reduced; particularly, the variation became so tiny for 2 h that it could be ignored. Therefore, the reaction time was fixed for 1.5 h. The acetylation reaction of cellulose was a heterogeneous reaction, which was a process from external reaction to internal reaction. Increasing the reaction time resulted in the sufficient contact between Ac_2_O and the inner hydroxyl groups of cellulose, which promoted the acetylation reaction of cellulose; thus, the mass and DS of RSCA increased. However, the cellulose and as-prepared RSCA were likely to degrade in the acidic condition, which resulted from the C_2_H_5_OH/H_2_O autocatalytic process of rice straw [36]. Therefore, 1.5 h was proved to be the optimal reaction time.

### 3.3. FTIR Spectrum

**Figure 5:**
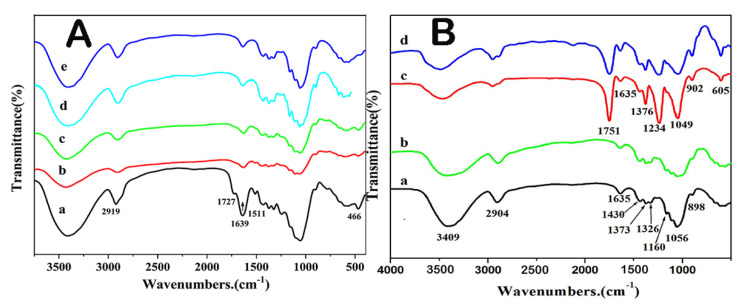
FTIR spectrum (**A**): (a) rice straw powder, (b) pretreated rice straw, (c) pretreated rice straw after secondary washing by C_2_H_5_OH/H_2_O solution, (d) rice straw-derived coarse cellulose, (e) RSC. FTIR spectrum (**B**): (a) RSC, (b) commercial cellulose, (c) RSCA, (d) commercial cellulose acetate.

The chemical structure of products from rice straw to rice straw-derived cellulose at different stages were characterized by infrared spectroscopy, as was shown in Figure 5A. Curve (a) was the infrared spectrum of rice straw powder; the characteristic absorption peaks at 3413 cm^−1^ and 2919 cm^−1^ were assigned to stretching vibration of -OH and the hydrocarbon stretching vibration of -CH_2_-, respectively. The characteristic absorption peak at 1727 cm^−1^ corresponded to the aliphatic ether group in hemicellulose [33], and the adsorption peak at 1511 cm^−1^ was indexed to the aromatic ring skeleton of lignin. For the curve (b) of pretreated rice straw in Figure 5A, it is detected that the characteristic adsorption peaks of 1511 and 1727 cm^−1^ were obviously weakened, which indicated that the C_2_H_5_OH/H_2_O autocatalytic process could remove most hemicellulose and lignin. Curve (c) displayed the IR spectrum of pretreated rice straw after secondary washing by C_2_H_5_OH/H_2_O solution; it is obviously detected that the characteristic peak at 1511 cm^−1^ furtherly weakened, because the adsorbed lignin on the surface of pretreated rice straw powder could be washed off when the pretreated rice straw powder was washed by the same concentration of C_2_H_5_OH/H_2_O. For curves (d) and (e) in Figure 5A, the characteristic peak of 1511 cm^−1^ belonging to lignin completely disappeared, which demonstrated that lignin was removed from the rice straw after alkali treatment and the H_2_O_2_ bleaching process.

FTIR spectrum of rice straw cellulose was presented in Figure 5B, the characteristic absorption peak at 3409 and 2904 cm^−1^ were corresponded to the stretching vibration of -OH and the hydrocarbon stretching vibration of -CH_2_-, respectively. The characteristic absorption peak at 1635 and 1373 cm^−1^ were assigned to the stretching vibration of -OH and blending vibration of O-H, respectively. A strong peak at 1056 cm^−1^ was detected, which was indexed into the skeletal vibration of the pyranose C-O-C [37], the peak at 898 cm^−1^ represented the characteristic adsorption of glycoside of the glucose unit. Moreover, there were spectral bands at 1430, 1326, 1160 and 1056 cm^−1^, all of which were corresponding to the characteristic adsorption of cellulose. Curve (b) was the infrared spectrum of commercial cellulose, and its characteristic peaks are almost identical to those of RSC, which proved that the purity of RSC was very high. Curve (c) and (d) were the infrared spectrum of RSCA and commercial cellulose acetate, respectively. It is obviously observed that all the characteristic peaks were almost the same. The absorption peak of curve (c) at 1751 cm^−1^ corresponded to the stretching vibration of C=O, the absorption peak at 1376 cm^−1^ was caused by the stretching vibration of C-H bond in the CH_3_COO- group, and the absorption peak near 1234 cm^−1^ belonged to the stretching vibration of C-O in the acetyl group. The enhanced absorption peak of C-O vibration in the C-O-C of the pyran ring skeleton was located at 1049 cm^−1^, and the weak absorption peaks at 1635 and 902 cm^−1^ were assigned to the β-glycosidic bond in the glycogen. The existence of these absorption peaks indicated that the RSCA was successfully prepared. Compared with the commercial products of cellulose and cellulose acetate, it is concluded that RSC and RSCA possessed very high purity.

### 3.4. XRD Patterns

**Figure 6:**
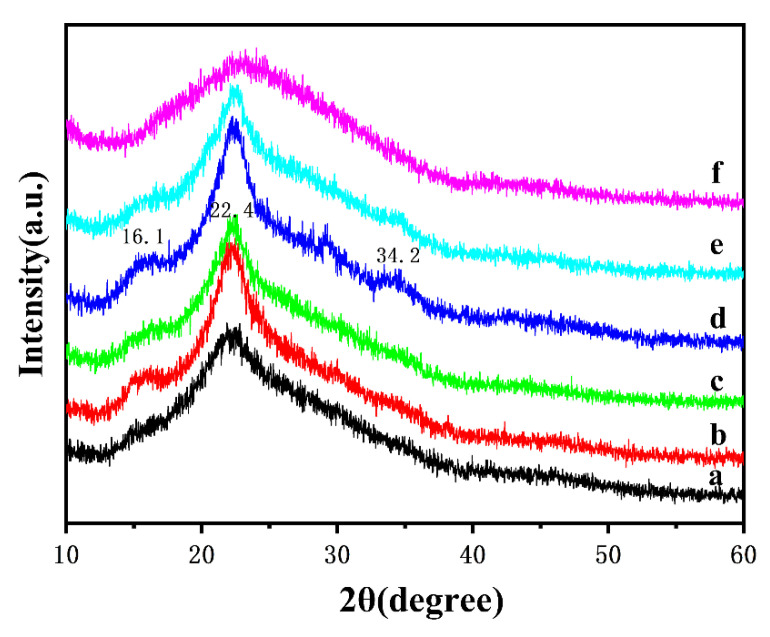
XRD patterns: (**a**) rice straw powder, (**b**) pretreated rice straw, (**c**) pretreated rice straw after secondary washing by C_2_H_5_OH/H_2_O solution, (**d**) rice straw-derived coarse cellulose, (**e**) RSC, (**f**) RSCA.

XRD patterns of products from rice straw to RSCA at different stages were depicted in Figure 6, and the characteristic diffraction peak at 22.4° was detected for all the curves in Figure 6, which was assigned to the typical lattice characteristic peaks of cellulose I_β_ [38]. As the extraction processes went on, a peak at 16.1° appeared, which was indexed to the typical lattice characteristic peaks of cellulose I_α_ [39]. This was mainly because the amorphous components such as the lignin and hemicellulose were removed, and the crystalline areas of cellulose were more exposed [40]; as a result, the crystallinity of RSC was enhanced. As seen from Figure 6d, a new characteristic peak near 34.2° appeared for the rice straw-derived coarse cellulose, which proved that the residual hemicellulose and lignin in the pretreated rice straw could be furtherly removed by dilute alkali treatment. For Figure 6e, the characteristic diffraction peaks located at 16.1°, 22.4° and 34.2° were all detected for RSC, and it is suggested that the crystalline form of RSC still belonged to the cellulose I structure after the H_2_O_2_ bleaching process of rice straw-derived coarse cellulose. Compared with Figure 6e of RSC, the RSCA in Figure 6f possessed the typical lattice characteristic peaks of cellulose I_β_, and the characteristic diffraction peak at 23.4° was distinctly weakened; moreover, the characteristic diffraction peak at 16.1° and 34.2° completely disappeared, which indicated that the crystallinity of rice straw-derived cellulose was weakened. This was mainly because of the reaction of the hydroxyl group with acetic anhydride during the esterification process, which resulted in the destruction of hydrogen bonds between molecules.

### 3.5. Morphology of Products from Rice Straw to RSCA

**Figure 7:**
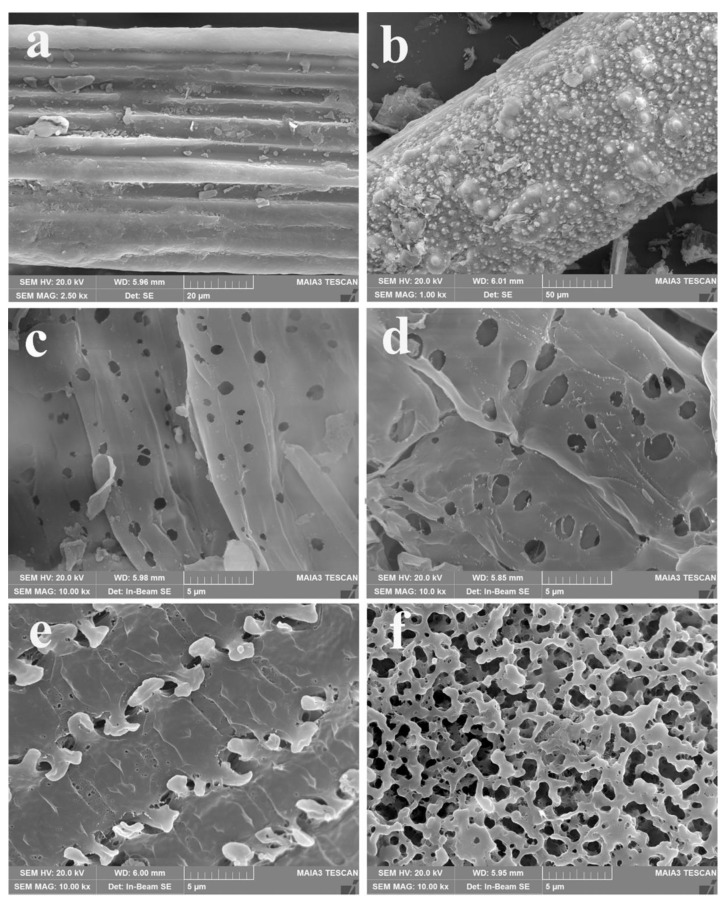
SEM images: (**a**) rice straw powder, (**b**) pretreated rice straw, (**c**) pretreated rice straw after secondary washing by C_2_H_5_OH/H_2_O solution, (**d**) rice straw-derived coarse cellulose, (**e**) RSC, (**f**) RSCA.

The morphologies of products from rice straw to RSCA at different stages were systematically characterized by SEM. As seen from Figure 7a, the rice straw powder with many stripe trenches displayed a fibrous appearance, and there were many solid particles of lignin and hemicellulose on the surface. As shown in Figure 7b, more spherical particles of absorbed lignin deposited on the surface of pretreated rice straw, which indicated that the dissolved lignin could be effectively removed from the rice straw by means of the C_2_H_5_OH/H_2_O autocatalytic process. When the pretreated rice straw was secondary washed by C_2_H_5_OH/H_2_O solution, the absorbed lignin on the surface of the pretreated rice straw could be effectively removed; meanwhile, the dissolution of lignin inside the cellulose and the degradation of hemicellulose resulted in the generation of numerous holes on the surface of the fiber, which is vividly displayed in Figure 7c. Spherical particles disappeared, and many holes with the pore diameter of 250–1000 nm appeared on the surface. When the dilute alkali treatment process was employed to prepare the rice straw-derived coarse cellulose, it is detected that the pores became bigger with the pore diameter of 500–2000 nm in Figure 7d, which demonstrated that the residual lignin could be furtherly removed in the form of alkali-soluble lignin. When the rice straw-derived coarse cellulose was refined to prepare the RSC by the H_2_O_2_ bleaching process, the morphology changed significantly in Figure 7e. Particularly, it is interesting that the RSCA possessed a multi-porous network structure in Figure 7f, which was ascribed to the acetylation reaction between the Ac_2_O and hydroxyl groups of the cellulose.

### 3.6. Solubility Analysis of the RSCA

**Figure 8:**
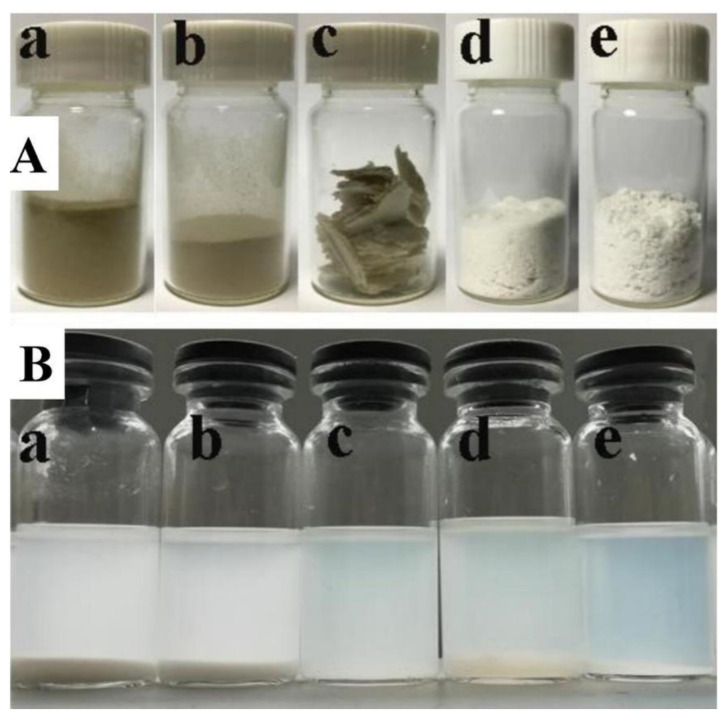
(**A**) Photographs of products from rice straw to RSCA at different stages: (a) rice straw powder, (b) pretreated rice straw, (c) rice straw-derived coarse cellulose (d) RSC, (e) RSCA. (**B**) Photographs showing the solubility property of RSCA with different DS: (a) DS = 0.51, (b) DS = 0.75, (c) DS = 1.12, (d) DS = 2.75, (e) DS = 2.82.

Products from rice straw to RSCA at different stages were vividly displayed in Figure 8A; it is examined that the color of the samples gradually changed from gray to white, and the apparent morphology gradually changed from powder to fluffy powder. In order to investigate the spinnability of cellulose acetate, a series of RSCA with different DS were successfully prepared, and the solubility analysis of cellulose acetate in methanol/dichloromethane solution was carried out, as displayed in Figure 8B. It is noted that the solubility of RSCA in methanol/dichloromethane solution distinctly boosted with the increasing DS value, the solution gradually became clear, and the insoluble matter obviously decreased, which was consistent with the results of insoluble rate shown in Figure 4A. It is proved that the highly purified RSC and RSCA were prepared in the paper.

### 3.7. Investigation the Spinnability of RSCA for Electrospinning

**Figure 9:**
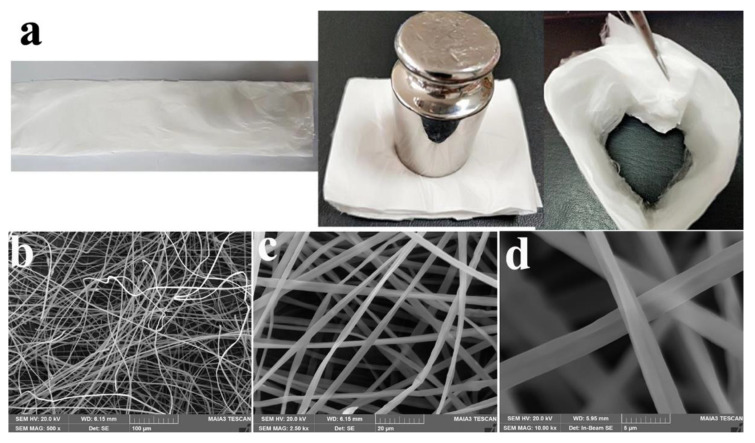
(**a**) Optical photographs showing the as-spun RSCA fibrous membrane. (**b**–**d**) SEM images of RSCA fibrous membrane.

As seen from Figure 9a, it is obviously noted that as-spun RSCA fibrous membrane could be easily folded, unfolded and curled, which demonstrated that the RSCA fibrous membrane was flexible. Furthermore, as shown in Figure 9b–d, the electrospun RSCA fibers displayed a smooth surface with a uniform diameter of approximately 2.5 μm, which could be adjusted by varying the spinning solution and electrospinning parameters, and it is concluded that the as-prepared RSCA was suitable for electrospinning. RSCA fibrous membrane has potential applications in medical materials, food packaging, nursing, catalysis, environmental pollution control and so on.

## 4. Conclusions

A novel method of extracting high-purity (92%) RSC from the rice straw was proposed, and a variety of RSCA with different DS values was prepared by the acetylation reaction. The optimal extraction conditions of RSC and preparation of RSCA were determined. The spinnability of electrospinning for RSCA was investigated in terms of two aspects: On the one hand, the solubility analysis of RSCA indicated that the as-prepared RSCA with a DS value of 2.82 possessed the best solubleness. On the other hand, RSCA was utilized as the raw material of spinning solution, and the flexible RSCA fibrous membrane was easily fabricated by the facile electrospinning method. Thus, it is demonstrated that as-prepared RSCA was suitable for electrospinning. Our proposed method in the paper provided a novel and detailed strategy for realizing the high-value utilization of waste rice straw resource; simultaneously, the environmental pollution resulting from the discarded rice straw was eliminated.

## Data Availability

The data presented in this study are available on request from the corresponding author.
